# Retracted: Correlation between Serum Levels of High Mobility Group Box-1 Protein and Pancreatitis: A Meta-Analysis

**DOI:** 10.1155/2019/5190178

**Published:** 2019-07-17

**Authors:** BioMed Research International

*BioMed Research International *has retracted the article titled “Correlation between Serum Levels of High Mobility Group Box-1 Protein and Pancreatitis: A Meta-Analysis” [1]. This article is one of a series of very similar meta-analyses written by different authors that were published in 2014 and 2015, characterized by searching the complementary and alternative medicine database CISCOM although the topic was not about complementary and alternative medicine [2]. The articles have the same structure, with the figures in the same order. The appearance of the figures and parts of the text are also similar.

We also found that the authors note there is publication bias, but they do not account for this in their analysis or discussion. The skew in Figure 6 is quite marked, meaning that negative results were likely not published. This may mean that the association of HMGB1 with pancreatitis is a false positive.

In addition, we checked the original files submitted by the authors and found that the files were edited by MedChina, a company previously alleged to be involved in the sale of articles [3].

The authors did not respond to our queries and we have informed the authors' institutions of our concerns.
